# Polycrystalline Superalloy Membranes Produced by Load-Free Coarsening of Incoherent γ′-Precipitates: Microstructure Evolution and Mechanical Properties

**DOI:** 10.3390/ma14040784

**Published:** 2021-02-07

**Authors:** Christian Voelter, Joachim Rösler

**Affiliations:** Institut für Werkstoffe, Technische Universität Braunschweig, Langer Kamp 8, 38106 Braunschweig, Germany; j.roesler@tu-braunschweig.de

**Keywords:** nickel-based superalloys, nanoporous membrane, load-free coarsening, porous materials, metallic membrane

## Abstract

Nanoporous superalloy membranes are a functional extension of the use of nickel-based alloys. The material, which is usually used for high-temperature applications, consists mainly of the two phases γ and γ′. Through coarsening of the precipitates and thus forming of a bicontinuous γ/γ′ network, membranes can be produced by removing either of these phases. From the single-crystalline alloy CMSX-4, the bicontinuous network can be formed either thermo-mechanically by directional coarsening of coherent precipitates or by load-free coalescence of incoherent precipitates. Recent investigations have shown that membranes also can be produced from polycrystalline starting material in both ways. In this article, the process route for membranes by load-free coarsening of incoherent γ′ precipitates from a carbon-free version of the polycrystalline alloy Nimonic 115 is presented. This manufacturing method has the advantage of its simplicity and in comparison to single-crystalline membranes it can be realized in larger scales. We discuss the microstructure and show the mechanical properties by means of tensile tests. Despite the grain boundaries as a mechanical weak link, polycrystalline membranes show promising mechanical properties. Their strength even exceeds that of the single-crystalline membranes despite the significantly higher pore volume content.

## 1. Introduction

Nanoporous superalloy membranes extend the use of nickel-based superalloys with regard to functional applications. The alloys typically used as high-temperature material show the phenomenon of rafting [1], especially when used as turbine blades where the material is exposed to high temperatures and stresses. As consequence the high amount of γ′-phase coarsens in a directed manner. This phenomenon was first used by [2] to produce metallic membranes, realizing that it leads to a bicontinuous γ/γ′-network so that selective leaching of one phase results in a membrane with open porosity at the location of the removed phase.

The processing route reproduces the rafting using creep tests with single crystalline superalloys. That means, as in the service of turbine blades, the material is at high temperatures under low stresses. Hence, a uniaxial stress is added to the internal coherency stresses due to misfit between γ and γ′ and plastic deformation of the γ-phase occurs. Due to different dislocation arrangements in horizontal and vertical γ-channels, the stress states are now no longer the same. This is followed by an elimination of the energetically less favorable matrix channels due to directional growth of the γ′ precipitates, leading to a bicontinuous γ/γ′-network. [3] Membranes produced this way might be used for filtering of submicron particles and microorganisms [4] or to produce nanoemulsions by premix membrane emulsification [5]. Furthermore, it was shown that besides rafting by a thermomechanical process also load-free coarsened membranes can be produced from CMSX-4 [6,7,8]. This simplifies the processing route since only a heat treatment is necessary to get a bicontinuous γ/γ′ structure. Depending on the heat treatment protocol, the bicontinuous network may be obtained by growth of coherent [6,7,8] or incoherent γ′-precipitates [6,7].

Recent investigations have shown that membrane fabrication is also viable with polycrystalline nickel-based superalloys by repeated steps of rolling and ageing, replacing creep deformation as the thermomechanical process to drive directional growth of the coherent γ‘-precipitates [9]. This leads to the fact that costs can be further reduced and also makes larger dimensions possible. Initial tests on polycrystals have shown that even load-free coarsening of incoherent γ′-precipitates is possible to produce polycrystalline membranes [10,11]. Rösler [3] gives an overview of processing routes, with specific microstructures, properties, advantages, and limitations.

Based on the results in [10,11], polycrystalline membranes made from 115NC were produced by load-free coarsening of incoherent γ′ in [3]. There, an already coarse initial structure was further coarsened for 288 h until an interpenetrating γ/γ′ structure was formed. It is shown that completely porous γ membranes can be produced by selective chemical extraction of the γ′-phase. However, neither an account on the microstructural evolution during processing nor information on the resulting mechanical properties is given in [3].

The present paper examines the microstructure evolution along the complete processing route for membranes obtained from polycrystalline superalloys by load-free coarsening of incoherent γ′-preciptates. (Note: We refer to the precipitates as incoherent due to the distinction between those two different coarsening methods, even though interfacial dislocations still may be present. While in coherent precipitates we have a growth mechanism controlled by coherency stresses, here we have loss of coherency and a diffusion controlled growth mechanism.) The microstructural analysis shows the morphology of the precipitates and their interconnection points. Furthermore, we present mechanical properties based on tensile tests with optical strain analysis and consider the fracture surfaces with regard to the failure mechanisms.

## 2. Materials and Methods

As base material we used slightly modified Nimonic 115 by removing carbon (Ni-14.4Cr-13.2Co-5Al-3.8Ti-3.3Mo-0.05Zr-0.044Mg-0.02B; wt.%). The alloy, referred to as 115NC, was produced by plasma arc melting (Lichtbogen-Schmelzanlage type 43022, PINK GmbH Vakuumtechnik, Wertheim, Germany) and cast into plates with dimensions of 60 mm × 60 mm × 3 mm. In order to get a thin sheet for the production of membranes, the plates were heat treated at 1473 K for 10 min, cooled down to 1173 K with 1 K/min and finally air cooled (AC). Afterwards, we cold rolled the plates in steps of 7% up to an overall thickness reduction of around 30% followed by the aforementioned heat treatment. This process was repeated until a sheet thickness of 0.5 mm was achieved. For the evaluation of the two-phase microstructure, without further processing into a porous membrane, the material was not rolled but used as cast. All heat treatments were performed in Carbolite Type CWF 13/23 chamber furnaces (Carbolite Gero GmbH, Neuhausen, Germany).

As explained in Section 1, it is possible to obtain a bicontinuous γ/γ′ microstructure by incoherent growth of γ′. For this purpose, the sheet respectively the plate is heat treated in three steps. After 1473 K for 10 min it is cooled down with 1 K/min to 1373 K and then air cooled. Afterwards, the precipitates were further coarsened at 1293 K/4 h/AC and 1273 K/144 h/AC.

Further preparation and methods depend on the purpose of investigation. To analyze the microstructure of the material, one specimen each was cut off and mounted for a cross section respectively surface grinding. After grinding with abrasive silicon carbide grinding papers (grit P240, P400, P600, P1200, P2500) and polishing with diamond suspension (9 µm, 6 µm, 3 µm, 1 µm) and oxide polishing suspensions with colloidal silica oxide (0.025 µm) they were etched with molybdic acid etchant (100 mL dest. H_2_O, 100 mL HNO_3_ (65%), 100 mL HCl (37%), 3 g MoO_3_ powder) for 4 s. The microstructure was characterized by scanning electron microscope (SEM; Zeiss LEO 1550, Carl Zeiss AG, Oberkochen, Germany).

During the heat treatment, a bicontinuous network of γ and γ′ should be formed. To receive a porous membrane, the following steps were performed.

First, the oxide layer resulting from heat treatment in air, along with a γ′-depleted zone beneath it, was remove by grinding leading to a thickness of less than 350 µm. Afterwards, the γ′ phase was selectively extracted with diluted molybdic acid etchant consisting of two parts distilled water, one part nitric acid, one part hydrochloric acid and 0.75 wt.% MoO_3_ powder [12]. The specimens were initially etched for 4 h while stirring. If the alcohol drop test (see below) showed that the specimens were not porous, an additional etching of 3 h was performed.

Cross sections of the membrane were mounted in epoxy resin and polished. Using an optical microscope (Zeiss Axio Imager 2, Carl Zeiss AG, Oberkochen, Germany), the membrane is examined for massive, i.e., unetched areas. Especially lens-shaped massive areas in the middle of the sample indicate incomplete extraction. In addition, the permeability of the membrane was investigated by using an alcohol drop test, which is a quick validation whether the membrane is porous throughout its thickness or not by pipetting an alcohol drop on the surface of the specimen. If it gets visible on the bottom, it can be assumed that an open porous structure has been formed. Note that this is only a qualitative method and no specific weight of alcohol or testing time is used.

For the tensile tests, five samples with 70 mm × 7.5 mm and a thickness between 325 and 335 μm were produced. Before selective phase extraction, the ends (grip section) were coated with a protective lacquer to prevent permeation of the etchant. The uncovered length is around 30 mm and the selective extraction of the γ′ phase was performed with a total time of 7 h as described above. Afterwards, the membranes were sprayed with a stochastic pattern for optical strain analysis (gom ARAMIS 5M, GOM GmbH, Braunschweig, Germany). For the spay pattern, the specimen were primed with a white developer for dye penetrant testing and afterwards black dots were randomly sprayed on with an air brush. Note that we use specimen with parallel edges instead of a dog bone design. This is discussed in Section 3.2. The tests were performed with a universal testing machine (Zwick/Roell Zmart.Pro, Zwick Roell AG, Ulm, Germany, 1 kN load cell) with a constant speed of 0.2 mm/s. The ARAMIS system is a contact-free, optical 3D measurement system that determines the displacement of defined measuring points on the surface of the specimen in individual images and calculates thus the deformation [13].

**Figure 1:**
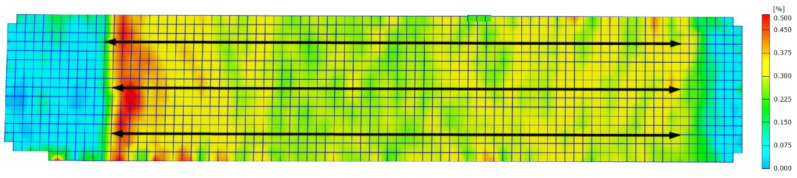
Visualization of the strain in loading direction in specimen 5 for the last frame before the fracture. Three lines each were defined to obtain the strain in the area of the porous structure. Using the frame with the largest elongation (last frame before fracture), the transition between grip section and porous structure is best distinguished.

For the evaluation of the tensile tests, the strain in loading direction was determined on the basis of the optical measurement results. For this purpose, only the fully porous areas are of interest. The endpoints of three parallel lines each specimen were used to obtain three independent measurements of the strain in the completely etched area, i.e., the area not covered by the lacquer. In the last frame before the fracture the transition between grip section and porous structure is best distinguished. Since the size of the etched area is also known (7.5 mm × 30 mm), the measuring points can be set as illustrated in Figure 1. To receive the total strain, the three measurements of the strain are averaged.

The membrane and fractured surface again were examined by SEM (Zeiss LEO 1550 and Hitachi TM3000, Hitachi Ltd., Tokyo, Japan).

### 2.1. Microstructural Analysis

#### 2.1.1. Determination of Pore Volume Fraction

**Figure 2:**
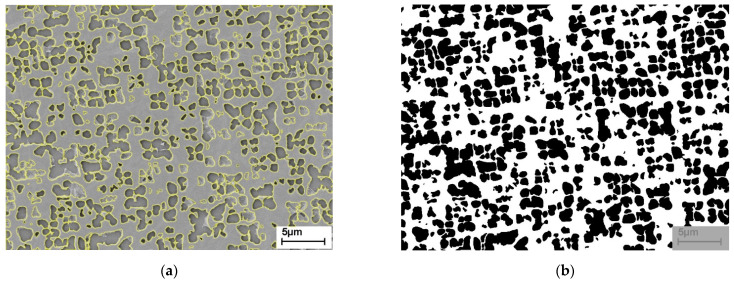
γ/γ′ microstructure: (**a**) outline of the γ′-precipitates drawn in ImageJ; (**b**) binarized microstructural image where the γ′-precipitates are black and the γ-matrix is white.

The pore volume fraction of the membranes was estimated by determining the γ′ volume fraction after the precipitates were coarsened in three SEM images. For this purpose, the contours of the γ′ particles were outlined by hand in the image processing program ImageJ (Figure 2a). Afterwards, these outlines were filled to create a binary black and white image, where precipitates (pores) are black and the matrix is white, see Figure 2b. The grey box is the area where the scale was, so this area was left out for further determination of the volume fractions. The area fractions of the black respectively white areas were displayed in ImageJ as values for the pore respectively matrix volume fraction.

#### 2.1.2. Determination of the Ligament Content at Grain Boundaries

**Figure 3:**
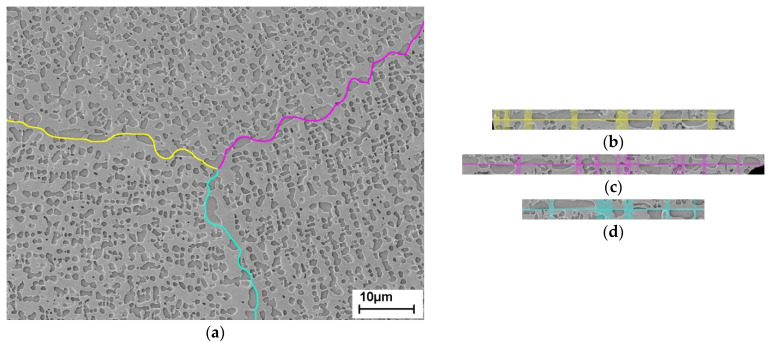
(**a**) Segmented lines on the grain boundaries of the aged material; (**b**) in ImageJ straightened grain boundary along the yellow, (**c**) magenta and (**d**) cyan segmented line with highlighted ligaments, i.e., those areas were on both sides of the grain boundary γ-matrix is present. The scale is valid for all images.

Based on five microstructural SEM images, of which three had triple junctions, the proportion of γ ligaments at grain boundaries was determined. These ligaments ensure that two adjacent grains are held together in the membrane. Figure 3 shows the etched microstructure of the material after the precipitates were coarsened. In ImageJ the grain boundaries were traced with segmented lines, see (a). To receive (b), (c), and (d), those lines were straightened. On this straightened grain boundary, it is easy to measure the proportion of the overall ligament length, i.e., those segments where γ is present in both grains, to the total length. This proportion corresponds with the percentage of ligament area over the whole grain boundary. From each triple point, the three grain boundary segments are considered individually, although each segment represents only a part of the whole grain boundary. Assuming that each grain boundary has a uniform distribution of precipitates, eleven individual grain boundaries can thus be included in the determination of the ligament content, regardless of the length of the segments considered.

**Figure 4:**
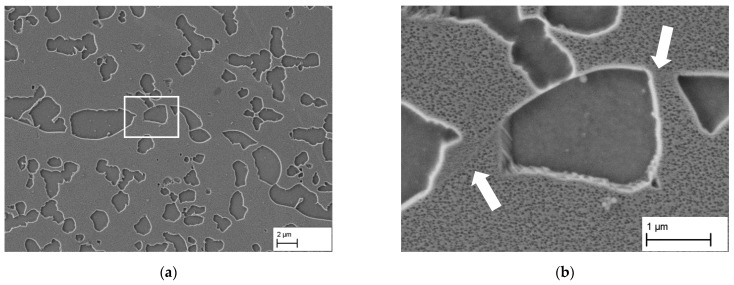
(**a**) Serrated grain boundary after slow cooling (0.25 K/min) from 1473 K. (**b**) Enlarged detail from (**a**), where the grain boundary within the matrix is clearly visible due to fine secondary γ′-particles formed during air-cooling after reaching 1373 K.

Figure 4 shows where to place the segments, i.e., where exactly the grain boundary is. In this figure the microstructure after slow cooling with 0.25 K min^−1^ from 1473 K to 1373 K is depicted. This heat treatment was chosen solely for reasons of easier understanding, since due to the very low cooling rate and without the following aging, the serration of the grain boundaries is more pronounced, and the grain boundary can be easily recognized due to small secondary γ′ precipitations from air cooling after the slow cooling. The serration of the grain boundaries are attributed by [14,15] to an increased solute diffusion along the grain boundaries, which causes the precipitates on the grain boundary side to grow faster.

The white arrows indicate the grain boundary on which coarse γ′ particles have been precipitated. Those particles show a smooth interface along the grain boundary and a rather rough one along the grain interior caused by the dislocations at the γ/γ′-interface. Similar observations were made by [14], those interfaces were determined as incoherent ones to the grain boundary respectively coherent ones to the parent grain. On the basis of this distinction, the path of the grain boundaries can also be well identified. In Figure 4, the γ-ligaments connecting adjacent grains can again be well seen.

## 3. Results and Discussion

### 3.1. Processing

The slow cooling (1 K/min) after solution heat treatment at 1473 K leads to a low nucleation rate of the γ′-phase and therefore few but big precipitates. This overaged γ/γ′ microstructure results in a low hardness of the material so that it is easy to roll.

**Figure 5:**
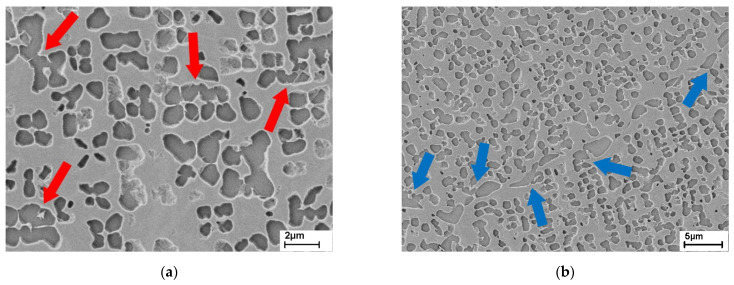
Bicontinuous γ/γ′ microstructure: (**a**) detail from Figure 2a: cross-linking of the octodendritic γ′-precipitates (red arrows); (**b**) detail from Figure 3a: grain boundaries with big precipitates and narrow ligaments of the matrix; blue arrows indicate links of the grain boundary precipitates to γ′ particles in the grain interior.

As the next step, the bicontinuous γ/γ′ microstructure is produced, using the heat treatment protocol mentioned in Section 2. In the microstructural images, Figure 5, the octodendritic morphology of the incoherent γ′ particles is clearly visible. As explained in [16], during slow, continuous cooling the γ′ morphology changes to octodendrites and later dendrites. Since the former cube corners grow preferentially, at these dendritic arms the precipitates coalesce and thus build a continuous network. The cross-links between two separately grown octodendrites are marked with red arrows in Figure 5a. They are relatively narrow. Consequently, the filter rating and permeability of the final membranes will be determined by the dimensions of these cross-links rather than the size of the relatively coarse octodendrites. Note that only a fraction of the cross-links present in three dimensions can be discerned in a 2D-section.

As shown in Figure 5b, the γ′ precipitates are significantly coarser at the grain boundaries than in the grain interior. This is a result of preferred nucleation at grain boundaries during slow cooling. Depending on the misorientation of adjacent grains, grain boundary precipitates vary in their size, i.e., big precipitates are found on large misorientations, the smallest on low angle and none at twin boundaries [14]. Around those precipitates in some cases bigger areas of γ are present. However, the arrows in Figure 5b show that the precipitates in the grain are connected to the precipitates at the grain boundaries.

**Figure 6:**
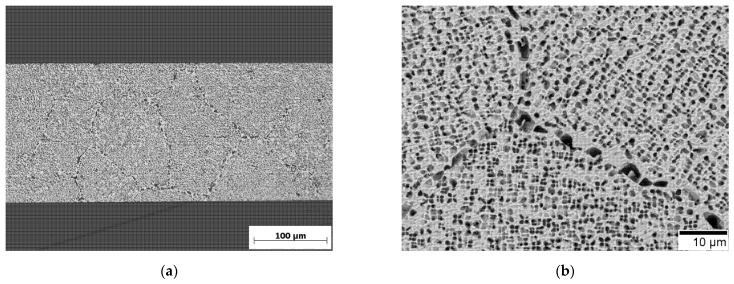
(**a**) Cross-section of the membrane in which the remaining γ matrix is white and the pores are black. (**b**) Grain boundary area on the surface of a tensile specimen (SEM with back scatter electron detector (BSE)).

In Figure 6a an optical microscope image of an etched membrane is shown. It is fully porous; no massive core remains. Since the whole cross-section is uniformly porous, the cross-links must occur not only occasional but at all precipitates, independent of their location in the grain or at the grain boundary, thus creating a bicontinuous structure. The permeability is also confirmed by an alcohol drop test.

As already illustrated in Figure 3 and Figure 4, Figure 6b clearly shows the narrow γ-ligaments between the voids (i.e., former γ′-precipitates) at the grain boundary that ensure the grains holding together. Certainly, those locations will lead to weak links regarding mechanical properties in the membranes. As explained in Section 2.1.2, the amount of precipitates at the grain boundaries was determined to estimate the amount of pores and ligaments at the grain boundary. As result an amount of about 15% ligaments was found, whereby the values vary between 5 and 22% (see Table 1). In contrast, the overall γ-content is about 56%. The related pore content was estimated on the basis of microstructural images as explained in Section 2.1.1. By means of three SEM images a pore volume of about 44% was determined, which corresponds to the phase content of γ′ calculated with the thermodynamic simulation software Thermo-Calc at 1273 K.

**Figure 7:**
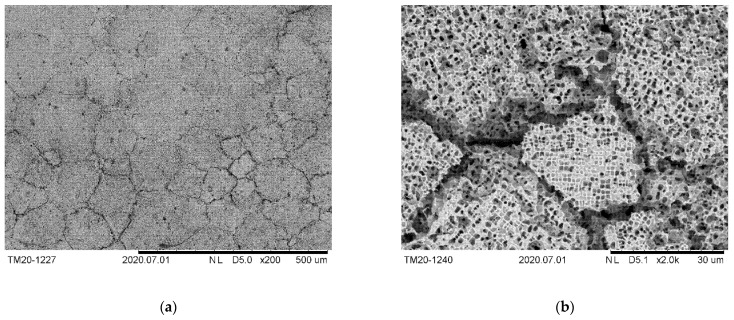
BSE-SEM images of the surface of a tensile specimen. (**a**) Transition area between perfect selectively etched structure and non-selective attack. (**b**) The damage occurs mainly at the grain boundaries.

This membrane shows that the process route is generally possible. However, there are occasional damages on the surface of the specimens, which are depicted in Figure 7. These damages are caused by a non-selective etching around the grain boundaries. In addition to the etching of γ′, a removal of the γ phase also took place, leading however only to quite shallow trenches. Still, it demonstrates a slight instability of the extraction process.

### 3.2. Mechanical Properties

In the tensile tests the mechanical behavior of the produced membranes was examined. For the results it is important to say that all values were related to the cross-section area of the whole porous structure and not the load-bearing cross section. Therefore, all parameters are marked with an asterisk (*).

**Figure 8:**
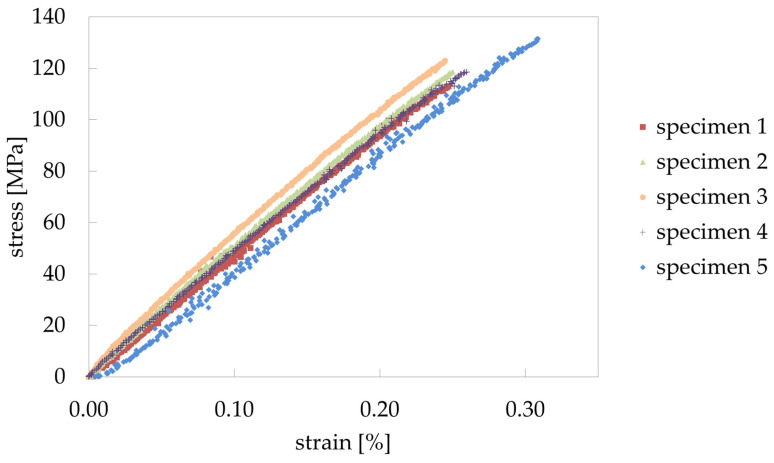
Stress-strain diagram of the tested samples. For specimen 1 and 2 all values incl. the unloading curves are shown.

The membranes have an average ultimate tensile strength of $R_{m}^{*}$ = 121 MPa and an average fracture strain of $A_{t}^{*}$ = 0.26%, whereby sample 5 differs most with $R_{m}^{*}$ = 132 MPa and $A_{t}^{*}$ = 0.31%. For determination of Young’s modulus, specimen 1 and 2 were unloaded again after reaching approx. 50 MPa, the elastic stiffness E* was then evaluated using the unloading curve. The value for both specimens is $E_{u}^{*}$ = 48 GPa. The stress strain diagram in Figure 8 shows macroscopically an essentially linear elastic behavior. This also can be seen when the elastic stiffness is estimated by E* = $R_{m}^{*}$/$A_{t}^{*}$ leading to an average value of 46.3 GPa for the five specimens. All values are listed in Table 2.

**Figure 9:**
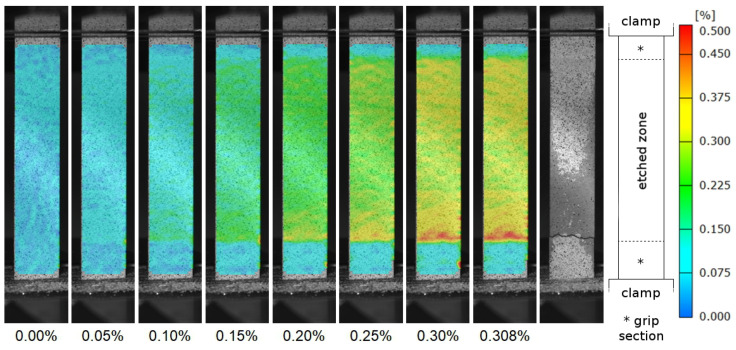
Strain distribution of specimen 5 measured by optical strain analysis. The nine subpictures show the states at the given total strain (values under the pictures) and, for the last, after the fracture. Values on the right side (color scale) show the local strain. The illustration on the right side shows in detail to the last two subpictures the exact position of the clamps and the transitions between etched zone and grip sections.

The strain distribution, depicted as exemplary for sample 5 in Figure 9, clearly shows that all membranes are deformed over their entire length and not only locally. Significantly lower strains can be seen in the grip section where the material is solid. This area is not considered further and is not included in any analyses (see Section 2). Note that the grip section refers to the former coated area and not the position where the clamps are attached. To illustrate this, grip section and end position of the clamps are marked in the right hand side of Figure 9. Although the coating prevents the acid from penetrating the surface, it does not prevent it from reaching inner areas through the uncoated area. This results in a slightly gradual transition from solid to porous and therefore a gradual increase in strain from solid to porous regions can be expected. This internal condition can be seen as compensation for an external dog bone design, both lead to a continuous increase of the cross section.

Differences between the examined samples can be seen for the area of the fracture. There, in specimen 5, the strain is slightly higher at every stage of the deformation. For all four other specimens, the fracture area either does not differ from the whole specimen or even shows slightly lower strains up to the last frame before fracture. For example, even in the last frame it is not obvious where the specimen is failing. Furthermore, in the area of sample 5 where fracture occurs, the difference between porous structure and grip section is clearly visible and very abrupt compared to the other specimens.

For specimens 1 and 2, the values for the strain at unloading and the second loading are essentially the same as for the first loading (in Figure 8 measuring points for loading and unloading are present). This is also shown by the strain distribution. The visualization at the three loading respectively unloading stages does not differ for the same strain.

All fractures occur in the uncoated area, i.e., in the reduced cross section when comparing to a dog bone shape. Nevertheless, it must be mentioned that with the exception of specimen 2, the failure occurs in the immediate vicinity of transition between former coated and uncoated area. However, only in specimen 5 the strain distribution is this abrupt as it can be seen in Figure 9. There can be various reasons for this. One might be an unselective etching inside the specimen caused by the coating or a stronger attack of the surface and thus stronger notch effect. Another possibility are stress concentrations due to the rather abrupt transition from porous to solid and the close proximity between porous section and the end of the clamped section. Due to these possible effects, the intrinsic strength of the porous material might be somewhat underestimated. However, we do not expect this influence to be large as the strength of specimen 2 is similar to the other ones (see Table 2).

Looking at the case where four out of five specimens fail in the transition area of the etched zone, strictly speaking these tests should be considered invalid in terms of determining the mechanical properties of the porous structure. It becomes clear that the internal increase of the cross-section does not seem to be sufficient and that the increase should be within the etched zone, such as in form of a dog bone specimen. However, as a lower estimation or the application case that membranes made in this way generally have a transition between porous and solid and thus this weak point, the results in our view provide relevant information about the mechanical properties.

**Figure 10:**
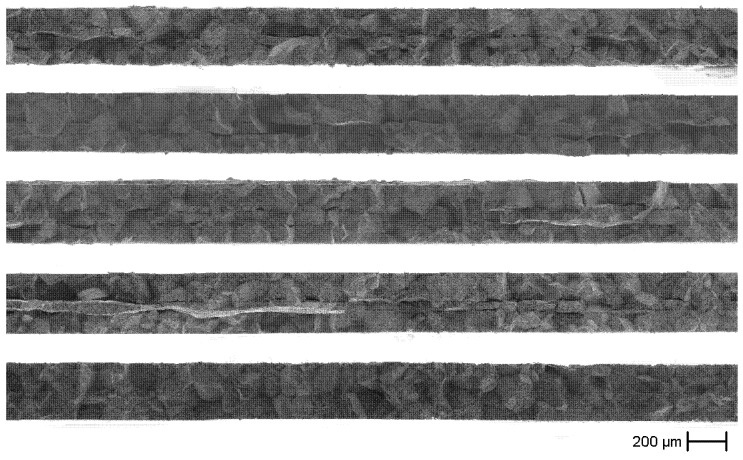
Fracture surfaces after tensile tests, from top to bottom: specimens 1–5 (SEM). An intercrystalline fracture path is clearly visible. In the middle of specimens 1 to 4 damages perpendicular to the fracture surface are visible.

The fracture surfaces in Figure 10 show an intercrystalline fracture pattern over most parts of the specimens. Furthermore, specimens 1 to 4 have fracture paths over the whole cross section parallel to the surface shown in Figure 9, i.e., perpendicular to the main fracture path, in the core of the specimen. Based on similar findings in [12] and observations in other experiments, this can be attributed to a non-selective dissolution of both phases at this very locally limited depth. When the crack reaches this weakened zone, it follows this zone for a while before it kinks back in the direction of the main fracture path. How and especially when this dissolution occurs is under investigation.

Localized transcrystalline fracture patterns occur repeatedly on all fracture surfaces. These can be divided into three categories. The most frequent transcrystalline fractures occur in the area of the damage in the core of the specimen. In this area, as already mentioned, the fracture first follows the non-selectively dissolved zone parallel to the applied load. In order to get back to the main path, the fracture must pass through the core area. If there locally is no grain boundary or none with a suitable orientation, the fracture occurs transcrystalline. Further, it is still possible that a grain boundary is present and has a suitable orientation, but an adjacent area has higher stresses due to a smaller cross-section and therefore fails first.

A similar situation may occur with the second type, where fragments of grains remain at the intersection of several grains. Here, intercrystalline fracture requires a large change in direction of the fracture path. Apparently, it is then sometimes easier to avoid this and follow a transcrystalline path for a while.

Finally, grains are observed which fail completely transcrystalline. These are surrounded by several grains whose broken grain boundaries are all in a similar plane. During the evaluation of the γ-ligament portion along grain boundaries it became clear that there are also grain boundaries with high ligament content. If the change of the fracture direction is too large or adjacent grain boundaries have a high fraction of ligaments, the fracture resistance along the grain boundary is sufficient for the fracture path to pass through the grain.

**Figure 11:**
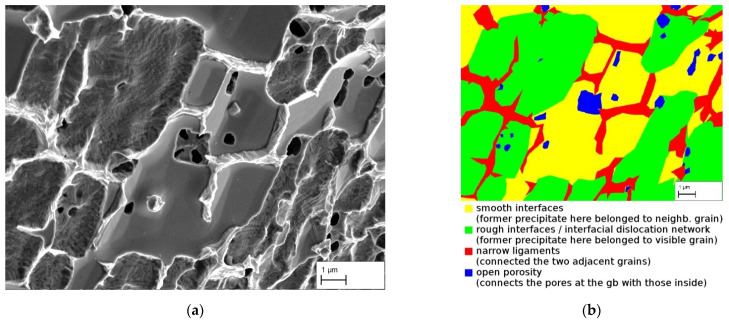
(**a**) Fracture surface of specimen 5 after the tensile testing (SEM image with InLens detector) showing deformed γ ligaments at a grain boundary. Octodendritic pores connect the coarse pores at the grain boundaries with those inside the grain. (**b**) Color map of that same surface to distinguish the different areas.

With further magnification it becomes clear that the grain boundaries are weakened due to the low amount of γ, as already stated in Section 3.1 on the basis of microsections. In Figure 11a the surface of specimen 5 after the tensile test is depicted. Since the fracture path is intercrystalline, it shows the surface of a single grain being situated on one side of the grain boundary. Four different areas can be distinguished, which are highlighted in Figure 11b. First, there are massive flat areas that are either smooth (yellow) or rough (green). After the heat treatment but before selective dissolution of the γ′-phase, coarse γ-precipitates were here. As already described in the course of determining the ligament fraction at grain boundaries, these precipitates have smooth interfaces on the grain boundary side and rough ones on the matrix side. These interfaces are revealed in Figure 11a as the γ′-particles were selectively dissolved. On the left side of Figure 11, the interfacial dislocation network of rough interfaces can be best seen. Here was a γ′-particle that belonged to the visible grain. The smooth surfaces are matrix of the shown grain to which precipitates of the neighboring grain were adjacent. Both cases result in the two grains not being connected to each other at those areas.

**Figure 12:**
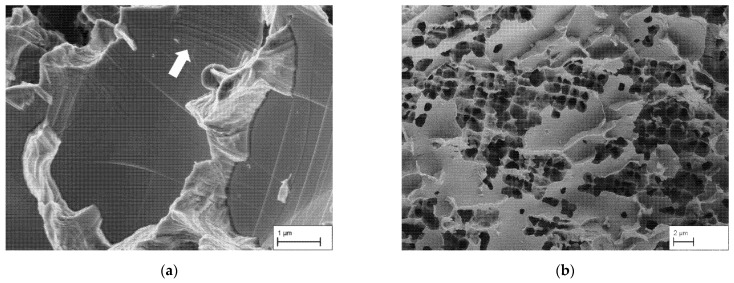
(**a**) Tapered ligaments with slip bands in the surrounding matrix on the fracture surface of specimen 2 (SEM image with InLens detector). (**b**) Fracture surface of specimen 5 (SEM image with Everhart-Thornley Detector for secondary electrons), visible here is the higher number of cross-links of pores from the inside of the grain to those at the grain boundary.

Between these large pores narrow ligaments connect the two grains. Those γ ligaments have tapered in the course of deformation and are finally broken in the form of narrow knife edges (red). Furthermore, in some cases plastic deformation continues even into the surrounding matrix of these ligaments and can be recognized as slip bands (white arrow in Figure 12a). This shows that the material behaves ductile. However, since plastic deformation is concentrated along the weak grain boundary areas, it is not visible in the stress-strain diagram, from which a macroscopically linear elastic behavior is deduced.

Finally, open porosity (blue) is visible. The octodendritic pores connect the coarse pores at the grain boundary with those from inside the grain and thus ensure permeability (compare with Figure 5b, blue arrows). This already was shown in Section 3.1 on the basis of microstructural images. At this particular grain boundary in Figure 11, only a few pores that reach into the grain are present. On other grain boundaries without the formation of massively covering γ′-precipitates, significantly more cross-links into the grain can be seen in Figure 12b.

Figure 11 shows, however, that despite the large precipitates and thus large pores, the characteristic pore size can be significantly smaller. In the grain interior and at the grain boundaries, it is often only the arms of the octodendrites that touch each other, resulting in pore diameters of often less than 1 µm. What kind of filter fineness this results in has not yet been further investigated.

In [17] the mechanical behavior of nanoporous superalloy membranes was analyzed. They were produced from single crystalline CMSX-4 by both creep deformation and load-free coarsening and electrochemical extraction. For the best microstructure obtained by creep deformation, an ultimate tensile strength of ~100 MPa was reached and an open porosity of 26% was reported. Considering the significantly higher porosity of 44% the strength levels observed here, even surpassing 100 MPa, are remarkable. In this context it has to be noted that single crystals and also the resulting single crystalline superalloy membranes are highly anisotropic in a number of respects. Firstly, the primary dendrites with [001]-orientation are essentially oriented in parallel to the growth direction of the single crystal. Between those dendrites small misorientations occur leading to small angle boundaries [18]. This disturbs the arrangement of the γ′-precipitates in such a way that weak fracture paths result in parallel to the primary dendrites when the single crystalline superalloy membrane is loaded perpendicular to the growth direction of the crystal [17]. In contrast, the length of dendrites is limited to the grain size in polycrystals and their orientation changes from grain to grain. Thus, such a weak fracture path extending throughout the sample cannot arise in a polycrystalline superalloy membrane. Secondly, tensile creep deformation of single crystalline superalloys such as CMSX-4 leads to pronounced directional γ′-growth perpendicular to the [001]-direction. When the resulting membrane is loaded in growth direction, the load is perpendicular to those ligaments, once again leading to a relatively weak fracture path [17]. In contrast, such a preferred γ′-orientation throughout the material does not occur in the polycrystalline superalloy membranes investigated here because (i) the crystallographic orientation changes from grain to grain and (ii) growth of incoherent γ′-particles is less regular. Even though polycrystalline superalloy membranes have their own weak fracture path as discussed above, it turns out that they are not inferior to those occurring in single crystalline membranes. This opens up an avenue for inexpensive production of strong, polycrystalline superalloy membranes.

Another frequently studied method of producing porous metals is dealloying. Here, porous structures with even smaller ligament diameters than in the membranes shown in the present article, down to a few 10 nm, are formed from mostly single-phase starting materials (often gold-based). [19,20,21,22,23,24,25] Due to significant volume shrinkage, large internal stresses and surface oxidation during the process, stress-corrosion cracking occurs, leading to the formation of extensive fabrication flaws [20,21,22]. This usually leads to poor mechanical properties, especially under tension, when the specimen dimensions are large enough to include these flaws. As mechanically studied specimen sizes are on a much smaller scale overall than shown here for compression and especially for tensile tests [23,24,25], these results are not comparable to those obtained here.

In [20], for nanoporous nickel, these cracks could be partially healed by using an ultrafine grained precursor alloy and post-dealloying annealing. During the heat treatment at 1173 K the ligament diameters grow from 15 nm to 450 nm. The tensile strength is anisotropic due to the rolling direction of the precursor material and reaches 150 MPa parallel and 94 MPa perpendicular to it. The specimens with a porosity of 53% obtain a fracture strain of 2.5%. However, these are also tensile specimens with a gauge length of 6 mm, a width of 1.2 mm, and a thickness of 30 µm.

## 4. Conclusions

It has been demonstrated that metallic membranes can be produced by the incoherent coarsening of γ′-precipitates in polycrystalline nickel-based superalloys. Pure thermal ageing leads to a bicontinuous γ/γ′-network and, after selective chemical extraction of the precipitate phase, to membranes with a pore volume fraction of 44%.

The mechanical properties were determined in tensile tests. An ultimate tensile strength of $R_{m}^{*}$ = 120 MPa and a Young’s modulus of $E_{u}^{*}$ = 48 GPa were obtained. The total strains measured by optical strain analysis are evenly distributed over the entire sample. Local increases in the area of the fracture are occasionally seen but are not the rule.

The fracture surfaces have shown that the grain boundaries are the mechanical weak links. Due to the formation of coarse γ′ precipitates at the grain boundaries and the resulting low ligament content of 15% on average, an intercrystalline fracture path is formed. Despite the macroscopically linear elastic stress-strain curve and the intercrystalline fracture path, a microscopically ductile fracture behavior is clearly visible on the fracture surfaces. The narrow matrix ligaments between the individual grains clearly show strong plastic deformation.

Because of the generally large precipitates, but narrow junctions between them, no concrete statement can be made regarding the filter fineness of the membranes by now. Further examination is necessary here.

The etching behavior also requires further research in order to find a reliable process for selective extraction. The limitation to the selective dissolution of one of the two phases is not always given. Both on the surface as well as in the core of the membrane there are occasional damages, which are expressed by partial dissolution of both phases. It stands to reason that the mechanical properties will further increase if the extraction process is improved.

Furthermore, the sample design should be investigated. Localization of the fracture near the transition to the solid area indicates less than ideal conditions here. Even if specimens with more central fractures do not show any deviation from the others, in general the transition area seems to be another weak point.

In conclusion, polycrystalline superalloy membranes have promising mechanical properties. Their process route and base material have significant advantages in terms of cost and their easy scalability to larger dimensions.

## Figures and Tables

**Table 1:** Amount of ligaments at the grain boundaries (lig. cont.). For every grain boundary (gb) only a part of it was evaluated. The length given here is of that evaluated segment.

		Length of gb [µm]	Lig. Cont. [%]
image 1 (triple point)	grain boundary 1	38.6	22.2
	grain boundary 2	31.0	20.5
	grain boundary 3	38.0	5.8
image 2 ^1^ (triple point)	grain boundary 4 ^1^	44.3	21.6
	grain boundary 5 ^1^	55.4	13.9
	grain boundary 6 ^1^	33.3	19.9
image 3 (triple point)	grain boundary 7	43.6	12.6
	grain boundary 8	35.1	4.7
	grain boundary 9	40.3	8.9
image 4	grain boundary 10	75.0	12.4
image 5	grain boundary 11	79.8	22.4

^1^ See Figure 3.

**Table 2:** Mechanical properties of the five tested specimen. $R_{m}^{*}$—ultimate tensile strength; $A_{t}^{*}$—fracture strain; E*—Young’s modulus estimated by $R_{m}^{*}$/$A_{t}^{*}$; $E_{u}^{*}$—Young’s modulus evaluated using the unloading curve.

	$R_{m}^{*}$ [MPa]	$A_{t}^{*}$ [%]	E* [GPa]	$E_{u}^{*}$ [GPa]
specimen 1	113.0	0.247	45.7	48.0
specimen 2	118.6	0.250	47.4	48.0
specimen 3	123.0	0.245	50.2	-
specimen 4	118.5	0.260	45.6	-
specimen 5	131.4	0.308	42.7	-
